# Effectiveness of Internet-Delivered Computerized Cognitive Behavioral Therapy for Patients With Insomnia Who Remain Symptomatic Following Pharmacotherapy: Randomized Controlled Exploratory Trial

**DOI:** 10.2196/12686

**Published:** 2019-04-11

**Authors:** Daisuke Sato, Naoki Yoshinaga, Eiichi Nagai, Kazue Nagai, Eiji Shimizu

**Affiliations:** 1 Department of Cognitive Behavioral Physiology Graduate School of Medicine Chiba University Chiba Japan; 2 Department of Rehabilitation Sciences Chiba Prefectural University of Health Sciences Chiba Japan; 3 Organization for Promotion of Tenure Track University of Miyazaki Miyazaki Japan; 4 Clinical Research Center Chiba University Hospital Chiba Japan; 5 Research and Education Center of Health Sciences Gunma University Graduate School of Health Sciences Maebashi Japan; 6 Research Center for Child Mental Development Chiba University Chiba Japan; 7 Cognitive Behavioral Therapy Center Chiba University Hospital Chiba Japan

**Keywords:** insomnia, cognitive behavioral therapy, randomized controlled trial, internet, benzodiazepines, residual symptoms

## Abstract

**Background:**

In reality, pharmacotherapy still remains the most common treatment for insomnia.

**Objective:**

This study aimed to examine the effectiveness of our internet-delivered computerized cognitive behavioral therapy (ICBT) program as an adjunct to usual care (UC) compared with UC alone in patients with insomnia who remain symptomatic following hypnotics.

**Methods:**

We recruited 23 patients with insomnia who remained symptomatic following pharmacologic treatment including benzodiazepines, and we conducted an exploratory randomized controlled trial. The primary outcome was the Pittsburgh Sleep Quality Index (PSQI) at week 6 of the treatment. Secondary outcomes were sleep onset latency, total sleep time, sleep efficiency, number of awakenings, refreshment and soundness of sleep, anxiety by Hospital Anxiety and Depression Scale, depression measured by the Center for Epidemiologic Studies Depression Scale, and quality of life (QOL) measured by the EuroQol-5D. All parameters were measured at weeks 0 (baseline), 6 (postintervention), and 12 (follow-up).

**Results:**

The adjusted mean reduction (−6.11) in PSQI at week 6 from baseline in the ICBT plus UC group was significantly (*P*<.001) larger than the adjusted mean reduction (0.40) in the UC alone group. Significant differences were also found in favor of ICBT plus UC for PSQI, sleep onset latency, sleep efficiency, number of awakenings, and depression at all assessment points. Refreshment, soundness of sleep, anxiety, and QOL improved by week 6 in ICBT plus UC compared with UC alone. There were no reports of adverse events in either group during the study.

**Conclusions:**

These results indicated that our 6-week ICBT program is an effective treatment adjunct to UC for improving insomnia and related symptoms even after unsuccessful pharmacotherapy.

**Trial Registration:**

University Hospital Medical Information Network Clinical Trials Registry: UMIN000021509; https://upload.umin.ac.jp/cgi-open-bin/ctr_e/ctr_view.cgi?recptno=R000023545 (Archived by WebCite at http://www.webcitation. org/75tCmwnYt).

## Introduction

### Background

Insomnia, which affects 10% to 12% of the total population, is characterized by the inability to fall asleep or awakening too early in the morning or during the night, resulting in nonrestorative sleep and decreased daytime functioning [1-4]. The spontaneous improvement of insomnia is low [5]. The treatment options for insomnia are psychotherapy and pharmacotherapy, and the American College of Physicians (ACP) recommends that all adult patients receive cognitive behavioral therapy (CBT) for insomnia as the initial treatment for chronic insomnia disorder. The ACP also recommends that clinicians use a shared decision-making approach, including a discussion with the patient, of the benefits, harms, and costs of the short-term use of medications before deciding whether to add pharmacological therapy in adults with chronic insomnia disorder in whom CBT for insomnia alone was unsuccessful [6]. In 2005, Vallières et al reported that pharmacotherapy before the initiation of CBT appears to be less effective than the combined treatment of pharmacotherapy plus CBT, followed by CBT alone [7]. Their study also revealed that the early introduction of CBT contributes to a maximization of the effect of pharmacotherapy.

Pharmacotherapy remains the most commonly used treatment option for insomnia worldwide [8]. In Japan, especially, CBT for insomnia is not covered by public health insurance at this time (2018), and thus the most common initial treatment for insomnia is a primary care physician’s prescription of an insomnia drug such as a benzodiazepine, nonbenzodiazepine, orexin receptor antagonist, or melatonin receptor agonist and antidepressants [9]. Although pharmacotherapy is associated with a high incidence of adverse effects including daytime sleepiness, recurrent insomnia, and drug dependence [10], physicians in Japan often prescribe excess doses of benzodiazepine [11]. A next step treatment in patients with insomnia who remain symptomatic following pharmacotherapy is strongly needed. Clinical practice guidelines suggest CBT, rather than pharmacotherapy, as the initial therapy for patients with insomnia [6,12,13]. Okajima et al showed that face-to-face CBT with a behavioral analysis is more effective than pharmacotherapy for Japanese chronic insomnia patients who are resistant to pharmacological treatment [14]. As Web-based programs are now more accessible and low cost and can be conveniently completed at one’s own time and place [15], we have developed an internet-delivered computerized cognitive behavioral therapy (ICBT) program for insomnia and we published a randomized controlled trial (RCT) design [16].

### Objectives

We conducted the RCT to examine the effectiveness of ICBT as an adjunct to usual care (UC) compared with UC alone, specifically targeting insomnia patients who remain symptomatic after pharmacotherapy. We hypothesized that among insomnia patients who remain symptomatic after pharmacotherapy, the augmentation with ICBT would be superior to UC alone in improving overall sleep quality, reducing anxiety and depression, and improving the patients’ quality of life (QOL).

## Methods

### Study Design and Participants

Our study protocol has been published [16] and is therefore only summarized here. This was a randomized controlled single-center trial conducted at the academic outpatient clinic of the Cognitive Behavioral Therapy Center of Chiba University Hospital between March 2016 and January 2018 as the recruitment period and between March 2016 and April 2018 as the trial period. Participants were recruited through posters and leaflets placed at medical institutions in the Chiba prefecture and through Web-based and newspaper advertisements. The inclusion criteria for this study were as follows: the participant regularly went to bed between 8 pm and 2 am; aged 18 to 65 years; having a primary diagnosis of insomnia according to the Diagnostic and Statistical Manual of Mental Disorders, Fifth Edition [1]; and the aforementioned insomnia remaining symptomatic. *Remaining symptomatic* was defined herein as having insomnia that is at least moderate in severity, based on a Pittsburgh Sleep Quality Index (PSQI) score of greater than 5.5 [15,17,18] after the use of hypnotics including nonbenzodiazepines, benzodiazepines, melatonin receptor agonists, orexin receptor antagonists, and antidepressants.

Each participant’s treatment history was conﬁrmed by their prescribing clinician and by chart review. All patients were evaluated by 2 researchers (a psychiatrist, ES, and a therapist, DS) who also verified the patient diagnosis and eligibility. They discussed the validity of the patient’s initial diagnosis and eligibility. Patients were reevaluated to cover important missing information based on suggestions derived from the discussion, and the ﬁnal diagnosis and eligibility were conﬁrmed by the 2 researchers.

The exclusion criteria included severe symptoms of anxiety or depression. Anxiety was assessed using the anxiety subscale of the Hospital Anxiety and Depression Scale (HADS) which contains 7 items. Depression was assessed using the total score of the Center for Epidemiological Studies Depression (CES-D) scale. Patients with a HADS score of greater than or equal to 10 or a CES-D score of greater than or equal to 30 were excluded. Patients with psychosis, organic mental disorder, or current high risk of suicide, substance abuse, or dependence within the 12 months before enrollment, antisocial personality disorder, or unstable medical condition were also excluded.

### Randomization

At the end of the baseline assessment, eligible participants were randomly assigned to either the UC arm or ICBT plus UC arm at a ratio of 1:1, with assignments made using the minimization method, ensuring a balance in baseline PSQI scores (PSQI<12) and gender. Each participant was then assigned to one of the 2 treatment regimes. Participants were blinded to the group to which they were assigned before consenting to participate in the study.

### Procedures

Primary physicians referred patients to the trial but continued to provide pharmacotherapy as UC to the patients in both groups, as described [19,20]. As part of the UC, both the UC only and ICBT plus UC groups received email magazines with general information about insomnia and hypnotics (in PDF format) by our research team 4 times over a 6-week period.

The ICBT program for insomnia was developed by one of the authors (ES) and is named the *Insomnia Improvement Internet Program*. The program is called IIIP (pronounced *three P*) for short, as III indicates the Roman numeral and *three P* sounds like *sleepy*.

The ICBT treatment consists of 5 weekly lessons and includes various elements that are commonly incorporated in face-to-face CBT for insomnia as follows: (1) keeping a sleep diary and understanding sleep hygiene; (2) changing sleep-related behaviors, including stimulus control; (3) restructuring distorted beliefs about sleep and sleep-related worries; (4) sleep restriction to increase sleep efficiency(SE); and (5) relaxation training, including breathing exercises and progressive muscle relaxation. Participants completed the 5 lessons over a 6-week period to provide sufficient time to become accustomed to the CBT. One of the authors (DS), a cognitive behavioral therapist, sent weekly emails to the participants to ask them about their homework and progress. The intervention was implemented as a cognitive behavioral therapist supported ICBT. Participants in the control group were offered the ICBT after the trial, if the UC did not make them sleep better.

### Outcomes

#### Primary Outcome

The primary outcome was the change in the PSQI score at week 6 from baseline (week 0). The PSQI is a self-rated questionnaire consisting of 19 questions across 7 subscales (sleep quality, sleep latency, sleep duration, habitual sleep efﬁciency, sleep disturbance, use of hypnotics, and daytime dysfunction). Each subscale is scored on a scale of 0 to 3. Subscale scores are summed to a total score ranging from 0 (good quality of sleep) to 21 (very poor quality of sleep). The PSQI was verified as a reliable and valid measure of subjective sleep quality in clinical practice and experimental research [17,18].

#### Secondary Outcomes

The secondary outcomes included the change in the PSQI score at week 3 and at week 12 from baseline (week 0) and the sleep onset latency (SOL), total sleep time (TST), SE, and number of awakenings (NA) extracted from the PSQI, as well as the current feeling of refreshment, perceived soundness of sleep (assessed by a visual analog scale), the anxiety subscale of HADS measuring anxiety, the CES-D score measuring depression, and EuroQol-5D (EQ-5D) score measuring the participant’s QOL [21].

The total score on the 7 HADS anxiety subscale items ranges from 0 (no symptoms of anxiety) to 21 (severe symptoms of anxiety). The total score on the 20 CES-D items ranges from 0 (no symptoms of depression) to 60 (severe symptoms of depression). We have described the 3-level version of the EQ-5D [21]. The EQ-5D [21] contains 5 items that assess QOL on a 3-point Likert scale ranging from 1 (not severe) to 3 (severe). The Japanese version of the EQ-5D was developed by Tsuchiya et al [22]. The EQ-5D is the most commonly used scale worldwide for calculating quality-adjusted life years (QALYs). QALYs are often used as the health outcome in cost-utility analyses and are typically estimated via an area under the curve analysis which involves summing the areas of the distribution shapes to calculate utility scores over the study period [23,24]. Our participants completed the HADS, CES-D, and EQ-5D questionnaires at home and sent them to us by email.

The therapist asked the participants about adverse event experiences at each assessment. All measures were assessed at weeks 0 (baseline), 3 (midintervention), 6 (postintervention), and 12 (follow-up).

### Statistical Analyses

The statistical analyses and reporting of this trial were conducted in accordance with the Consolidated Standards of Reporting Trials (CONSORT) guidelines. For baseline variables, summary statistics were constructed, using frequencies and proportions for categorical data and the mean and SD for continuous variables. Baseline variables were compared using Fisher exact test for categorical outcomes and the unpaired *t* test for continuous variables. For the primary analysis comparing treatment effects, the least-squares means, and their 95% CIs were estimated by an analysis of covariance (ANCOVA) with the change in total PSQI scores at week 6. This ANCOVA model took into account the variation caused by treatment effects, and the participants’ gender and baseline PSQI score were entered as covariates. Analyses of secondary outcomes were performed in the same manner as the primary analysis. All *P* values were 2-sided. *P* values less than .05 were considered significant. All statistical analyses were performed using SAS version 9.4 software (SAS Institute).

As described by our published design study [16], the sample size was based on a previous study by van Straten et al [25], which indicated that the estimated group difference in changes of PSQI scores from baseline was approximately 2.86 (ICBT group=3.00; control group=0.04). Assuming a group difference of 2.86 points (SD 2.5), 13 subjects per group will provide 80% power to detect a difference in PSQI scores between the UC arm and ICBT plus UC arm, using a 2-sided, 2-sample *t* test at a 5% significance level. Thus, allowing for a 10% dropout rate, 15 participants are required per group, for a total of 30 participants in the study.

### Ethical Approval

Written informed consent was obtained from all patients after the procedures had been fully explained. Ethical approval was obtained from the Institutional Review Board of Chiba University Hospital (no. G27040), and the trial was registered as UMIN000021509.

## Results

### Recruitment

**Figure 1:**
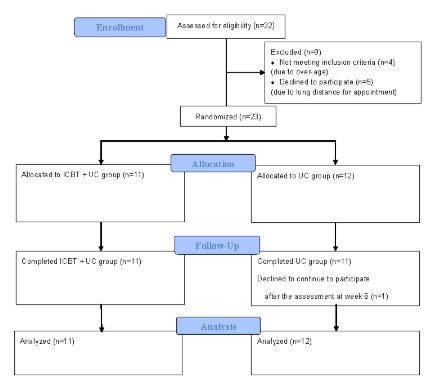
The CONSORT flow diagram for the trial. ICBT: internet-delivered computerized cognitive behavioral therapy; UC: usual care.

Figure 1 shows the patient recruitment flow diagram, based on the CONSORT guidelines. A total of 32 patients applied to participate through our website. Of the 9 patients who were excluded, 4 did not meet one of the inclusion criteria because of their age (over the limit) and 5 declined to participate because of the long distance to our hospital. A final total of 23 patients attended the face-to-face baseline assessment, and all 23 were enrolled in the study. We randomly assigned the 23 patients to the ICBT plus UC and UC groups. Furthermore, 1 patient in the UC group declined to continue to participate and dropped out from the study after the assessment at week 6. Though the originally planned recruitment rate would be 2 participants per month, the real average recruitment rate was 1 participant per month through posters and leaflets placed at medical institutions in the Chiba prefecture and through Web-based and newspaper advertisements within the planned recruitment period between March 2016 and January 2018. We had to stop recruitment after we entered 23 patients after 23 months of trial commencement on the closing date (see Figure 1).

### Demographics and Clinical Characteristics

Table 1 summarizes the baseline demographic and clinical characteristics of the participants. There were no significant between-group differences in any of the characteristics, including the number of patients with a baseline PSQI score less than 12 (PSQI<12) and the total PSQI score. The ICBT plus UC group received pharmacologic treatment including zolpidem (n=3), brotizolam (n=2), eszopiclone (n=1), etizolam (n=1), estazolam (n=1), flunitrazepam (n=1), alprazolam (n=1), and the combined use of zolpidem and brotizolam (n=1). The UC group received pharmacologic treatment including zolpidem (n=3), brotizolam (n=2), zopiclone (n=1), etizolam (n=1), the combined uses of zolpidem and etizolam (n=1); lormetazepam and flunitrazepam (n=1); quazepam and lorazepam (n=1); brotizolam, nitrazepam, and clonazepam (n=1); and zolpidem, lormetazepam, and trazodone hydrochloride (n=1). Thus, all 23 of the participants were taking one or more benzodiazepines. There was no change of use of sleep medication during intervention and follow-up period among the ICBT plus UC and UC group patients. We are conducting further study to estimate a difference in reduced use of sleep medication after the end of the follow-up period among the ICBT plus UC and UC group patients.

**Table 1:** Baseline characteristics (N=23).

Variable	ICBT^a^ + UC^b^ (n=11)	UC (n=12)
Female, n (%)	9 (81.8)	9 (75.0)
Age in years, mean (SD)	49.4 (13.8)	50.5 (8.8)
Material status (married of living as married), n (%)	9 (81.8)	10 (83.3)
Length of education in years, mean (SD)	14.1 (2.5)	14.5 (1.9)
Employment status (in paid employment, full or part-time), n (%)	9 (81.7)	9 (75.0)
Alcohol drinking (habitual or opportunity drinking), n (%)	3 (27.3)	7 (58.3)
Smoking, n (%)	0 (0)	0 (0)
Duration of insomnia in years, mean (SD)	6.3 (5.1)	6.0 (7.7)
Number of patients with PSQI^c^ less than 12, n (%)	3 (27.3)	3 (25.0)

^a^ICBT: internet-delivered computerized cognitive behavioral therapy.

^b^UC: usual care.

^c^PSQI: Pittsburgh Sleep Quality Index.

### Primary Outcome

**Figure 2:**
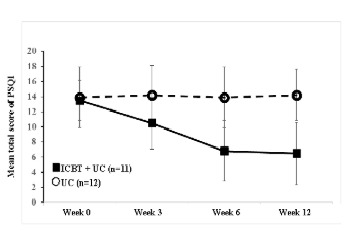
Mean and SDs (raw data) for the primary outcome, the Pittsburgh Sleep Quality Index (PSQI) score improvement. ICBT: internet-delivered computerized cognitive behavioral therapy; UC: usual care.

The raw data (ie, mean and SDs) of the participants’ PSQI scores at the 4 assessment points are shown in Figure 2 and Table 2. At week 6, the adjusted mean reductions in PSQI from baseline were −6.11 (95% CI −7.45 to −4.78) and 0.40 (95% CI −0.83 to 1.63) for the ICBT plus UC and UC groups, respectively. The group difference was significant at −6.51 (95% CI −8.15 to 4.87, *P*<.001; Table 3). The combination therapy, that is, ICBT plus UC, was therefore superior to UC alone.

### Secondary Outcomes

At week 3, the adjusted mean reductions in PSQI from baseline were −2.66 (95% CI −3.63 to −1.69) and 0.45 (95% CI −0.45 to 1.34) for the ICBT plus UC and UC groups, respectively. The group difference was significant at −3.10 (95% CI −4.29 to 1.92, *P*<.001; Table 3). At week 12, the adjusted mean reductions in PSQI from baseline were −6.40 (95% CI −8.05 to −4.75) and 0.44 (95% CI −1.11 to 1.99) for the ICBT plus UC and UC groups, respectively. The group difference was significant at −6.84 (95% CI −8.90 to 4.77, *P*<.001; Table 3). The combination therapy of ICBT plus UC was thus superior to UC alone at all 3 of the assessment time points.

The raw data (mean and SDs) and the adjusted mean changes for the secondary outcome measures are presented in Table 2 and Table 3, respectively. At week 3, compared with the UC group, significant improvements were observed in the ICBT plus UC group in the SOL, SE, NA scores, and CES-D (all *P*<.05). At week 6, compared with the UC group, significant improvements were observed in the ICBT plus UC group in the SOL, SE, NA, current feeling of refreshment, perceived soundness of sleep, the anxiety subscale of HADS, the CES-D, and the EQ-5D (all *P*<.05). At week 12, compared with the UC group, significant improvements were observed in the ICBT plus UC group in SOL, SE, NA, current feeling of refreshment, perceived soundness of sleep, the anxiety subscale of HADS, the CES-D, and the EQ-5D (all *P*<.05). There were no significant differences in the TST between the 2 groups at the 3 time points.

This result shows that, compared with the insomnia patients who received only UC, those who received ICBT plus UC reported significant improvements in their current feeling of refreshment, perceived soundness of sleep, measures of anxiety and depression, and functioning or QOL improved by week 6. The administration of ICBT improved the SOL, SE, NA, and depression at an early stage, but not the TST at the final stage.

**Table 2:** Raw data of the primary and secondary outcomes (N=23).

Variable		ICBT^a^ + UC^b^ (n=11)	UC (n=12)
**Primary outcome: sleep characteristics**			
	PSQI^c^, mean (SD)	13.5 (2.7)	13.9 (4.0)
**Secondary outcomes: sleep**			
	SOL^d^, minute, mean (SD)	54.5 (43.4)	41.7 (20.7)
	TST^e^, hour, mean (SD)	6.3 (2.1)	5.9 (1.2)
	Sleep efficiency, %, mean (SD)	73.2 (8.4)	73.6 (20.6)
	NA^f^, mean (SD)	3.5 (2.9)	2.9 (1.7)
	Refreshment, mean (SD)	3.8 (2.6)	4.3 (2.5)
	Soundness of sleep, mean (SD)	4.0 (2.7)	3.2 (2.2)
**Secondary outcomes: health**			
	Anxiety, HADS^g^, mean (SD)	4.8 (3.2)	6.7 (2.5)
	Depression, CES-D^h^, mean (SD)	18.5 (4.6)	21.4 (5.4)
	QOL^i^, EQ-5D^j^, mean (SD)	0.911 (0.125)	0.703 (0.174)

^a^ICBT: internet-delivered computerized cognitive behavioral therapy.

^b^UC: usual care.

^c^PSQI: Pittsburgh Sleep Quality Index.

^d^SOL: sleep onset latency.

^e^TST: total sleep time.

^f^NA: number of awakenings.

^g^HADS: Hospital Anxiety and Depression Scale.

^h^CES-D: Center for Epidemiologic Studies Depression Scale.

^i^QOL: quality of life.

^j^EQ-5D: EuroQol-5D.

**Table 3:** Adjusted mean changes in the primary and secondary outcomes.

Changes from baseline			ICBT^a^+UC^b^ (n=11)		UC (n=12)		Intergroup difference		*P* value
			Least squares mean	95% CI	Least squares mean	95% CI	Difference	95% CI	
Primary outcome, PSQI^c^, week 6			−6.11	−7.45 to −4.78	0.40	−0.83 to 1.63	−6.51	−8.15 to −4.87	<.001
**Secondary outcomes**									
	**PSQI**								
		Week 3	−2.66	−3.63 to −1.69	0.45	−0.45 to 1.34	−3.10	−4.29 to −1.92	<.001
		Week 12	−6.40	−8.05 to −4.75	0.44	−1.11 to 1.99	−6.84	−8.90 to 4.77	<.001
	**SOL^d^, minute**								
		Week 3	−10.82	−19.58 to −2.06	3.25	−5.00 to 11.49	−14.07	−24.97 to −3.16	.01
		Week 6	−27.01	−34.83 to −19.19	4.64	−2.73 to 12	−31.65	−41.39 to −21.91	<.001
		Week 12	−29.32	−38.90 to −19.74	6.47	−2.73 to 15.66	−35.79	−47.88 to −23.70	<.001
	**TST^e^, hour**								
		Week 3	0.49	0.13 to 0.86	0.07	−0.27 to 0.41	0.43	−0.03 to −0.88	.06
		Week 6	0.58	0.03 to 1.13	−0.08	−0.59 to 0.43	0.66	−0.02 to 1.34	.06
		Week 12	0.49	−0.08 to 1.05	−0.05	−0.59 to 0.48	0.54	−0.17 to 1.25	.13
	**Sleep efficiency, %**								
		Week 3	4.65	0.59 to 8.72	−3.72	−7.45 to 0.01	8.37	3.44 to 13.31	.002
		Week 6	13.24	8.67 to 17.81	−4.68	−8.87 to −0.48	17.92	12.37 to 23.47	<.001
		Week 12	13.32	8.68 to 17.97	−7.00	−11.37 to −2.63	20.32	14.55 to 26.1	<.001
	**NA^f^**								
		Week 3	−0.53	−1.17 to 0.12	0.61	0.01 to 1.21	−1.14	−1.93 to −0.34	.008
		Week 6	−2.04	−2.83 to −1.26	0.17	−0.55 to 0.89	−2.22	−3.18 to −1.25	<.001
		Week 12	−1.95	−2.81 to −1.08	0.05	−0.77 to 0.87	−2.00	−3.08 to −0.91	<.001
	**Refreshment**								
		Week 3	0.53	−0.30 to 1.35	−0.20	−0.95 to 0.56	0.72	−0.28 to 1.73	.15
		Week 6	2.09	0.67 to 3.51	−0.49	−1.79 to 0.81	2.58	0.84 to 4.31	.006
		Week 12	2.06	0.48 to 3.64	−1.03	−2.52 to 0.46	3.09	1.11 to 5.07	.004
	**Soundness of sleep**								
		Week 3	0.12	−0.82 to 1.06	−0.61	−1.49 to 0.27	0.73	−0.44 to 1.9	.20
		Week 6	2.76	1.25 to 4.28	0.30	−1.11 to 1.72	2.46	0.58 to 4.34	.01
		Week 12	2.70	1.21 to 4.18	−0.36	−1.77 to 1.06	3.05	1.15 to 4.96	.004
	**Anxiety, HADS^g^**								
		Week 3	−0.08	−0.74 to 0.58	0.68	0.11 to 1.26	−0.77	−1.58 to 0.04	.06
		Week 6	−0.83	−1.62 to −0.04	1.17	0.48 to 1.85	−1.99	−2.96 to −1.02	<.001
		Week 12	−0.95	−1.98 to 0.08	1.45	0.54 to 2.37	−2.40	−3.70 to −1.11	<.001
	**Depression, CES-D^h^**								
		Week 3	−2.46	−4.34 to −0.59	0.74	−1.00 to 2.47	−3.20	−5.56 to −0.84	.01
		Week 6	−5.18	−7.77 to −2.60	1.14	−1.25 to 3.53	−6.32	−9.57 to −3.07	<.001
		Week 12	−5.94	−8.92 to −2.96	2.02	−0.82 to 4.85	−7.96	−11.77 to −4.14	<.001
	**QOL^i^, EQ-5D^j^**								
		Week 3	−0.0065	−0.0516 to 0.0386	−0.0145	−0.0546 to 0.0256	0.0079	−0.0529 to 0.0688	.78
		Week 6	0.0487	0.0001 to 0.0972	−0.0793	−0.1225 to −0.0361	0.128	0.0625 to 0.1935	<.001
		Week 12	0.059	0.003 to 0.115	−0.0943	−0.1457 to −0.0429	0.1533	0.0759 to 0.2307	<.001

^a^ICBT: internet-delivered computerized cognitive behavioral therapy.

^b^UC: usual care.

^c^PSQI: Pittsburgh Sleep Quality Index.

^d^SOL: sleep onset latency.

^e^TST: total sleep time.

^f^NA: number of awakenings.

^g^HADS: Hospital Anxiety and Depression Scale.

^h^CES-D: Center for Epidemiologic Studies Depression Scale.

^i^QOL: quality of life.

^j^EQ-5D: EuroQol-5D.

For the screening of insomnia, 5.5 is considered the optimal cut-off score of the PSQI [26,27]. We therefore set the threshold at a PSQI score of 5.5 to determine the remission of insomnia. At week 6, 36% (4/11) of the patients in the ICBT plus UC group showed remission of insomnia with a PSQI score less than 5.5, whereas no UC group patient showed remission. At week 12, 45% (5/11) of the patients in the ICBT plus UC group and no patient in the UC group showed remission with a PSQI score of less than 5.5. The remission rates at week 6 and 12 were significantly higher in the ICBT plus UC group compared with the UC group by Fisher exact test (*P*<.05). There were no participants who could not complete the 5 lessons over a 6-week period in the intervention group and did not get back to a cognitive behavioral therapist’s weekly emails about their homework and progress. There were no reports of any adverse events in either group during the study.

## Discussion

### Principal Findings

This is the first RCT to examine the effectiveness of ICBT as a next step treatment for patients with insomnia who remain symptomatic despite drug treatment. Our findings demonstrate that the ICBT was effective as an adjunct to UC in reducing the severity of insomnia at week 6 immediately after the intervention. Moreover, the patients who received ICBT showed significant improvements at week 3 as a midpoint of the intervention and at week 12 of the follow-up period.

### Comparison With Prior Work

A meta-analysis of RCTs including 14 records of 15 studies (1013 experimental group participants and 591 waiting list participants) showed that internet-based CBT for adults with insomnia is an effective treatment [28]. Except for being insomnia patients who remain symptomatic following treatment with a hypnotic in this study, the baseline clinical characteristics (age and sex) of our recruited patients in Japan are similar to those in Western countries. In their study of patients recruited from the general population, van Straten et al [25] reported that their guided ICBT for insomnia changed the mean PSQI score from 12.4 (SD 2.1) at pretreatment to 8.9 (SD 2.6) at week 6 post-treatment compared with a wait-list control group’s score reduction from 11.7 (SD 2.2) to 11.6 (SD 2.5). In an investigation of patients with comorbid psychiatric diagnoses who were taking one or more psychotropic medications, Feuerstein et al [29] showed that their computer-based delivery of CBT for insomnia significantly improved the patients’ PSQI scores compared with an active control group (sleep diary group). Our present findings regarding PSQI improvement seem comparable to these 2 studies even though the patient populations differ.

A meta-analysis of RCTs including 87 RCTs comparing 118 treatments (3724 patients) to nontreated controls (2579 patients) showed that face-to-face and ICBT for adults with insomnia are effective treatments [30]. In addition, that meta-analysis described between-group effect sizes of outcomes concerning sleep as follows: insomnia severity index (Hedges g=0.98), SE (g=0.71), PSQI (g=0.65), wake after sleep onset (g=0.63) and SOL (g=0.57), NA (g=0.29), and sleep quality (g=0.40). The meta-analysis authors also mentioned that the smallest effect was on TST (g = 0.16) [30]. The various effect sizes on different sleep outcomes seem to be consistent with our finding that our ICBT program showed a remarkably large Hedges g value for PSQI (g=−3.36), SE (g=2.36), SOL (g=−1.80), and NA (g=−1.39), but not for TST (g=0.42); we calculated the between-group effect sizes at week 6 from baseline.

Moreover, Lancee et al [31] reported superior performance of face-to-face treatment relative to online treatment in their RCT comparing 3 conditions: guided online, face-to-face, and wait-list. In Japan, Yamadera et al [32] reported that face-to-face individual CBT for insomnia resulted in a PSQI improvement from 12.7 (SD 0.7) to 8.9 (SD 0.6) compared with the improvement because of group CBT from 12.2 (SD 0.5) to 10.1 (SD 0.7). Okajima et al [14] reported that face-to-face individual CBT for pharmacological treatment-resistant chronic insomnia resulted in PSQI improvement from 13.59 (SD 3.25) to 8.10 (SD 2.95) compared with the improvement because of UC from 12.45 (SD 2.52) to 11.17 (SD 3.23). Our observation of PSQI improvement from 13.5 (SD 2.7) to 6.8 (SD 4.0) versus the improvement with UC from 13.9 (SD 4.0) to 13.9 (SD 4.0) seems to be comparable to the above 2 Japanese studies of face-to-face treatment, even though we used a guided online program. Further research is necessary to compare online CBT with face-to-face CBT, including cost-effectiveness and patients’ preferences.

According to the algorithm in a new clinical practice guideline for the pharmacologic treatment of chronic insomnia in adults issued by the American Academy of Sleep Medicine [33], if pharmacologic treatment (short-term intermediate-acting benzodiazepine receptor agonists or ramelteon) does not improve the symptoms of an individual with chronic insomnia, clinicians should consider switching to another modality (ie, CBT) or combined treatment with CBT. The results of our present analyses suggest that the simple continuation of pharmacologic treatment was largely ineffective for our population of patients with insomnia and that clinicians should consider providing ICBT or referring patients to a CBT therapist if pharmacologic treatment is not sufficiently effective.

A meta-analysis of adherence to ICBT showed that the percentage of noncompleters of total ICBT intervention was 34.9% [34]. All participants in the ICBT plus UC group accomplished the total ICBT program in our study, one strength of this study is the low rate of dropout.

### Limitations

This study has the following 5 limitations. First, we were unable to elucidate specific effects of the ICBT program because a psychological placebo group was not used to control for nonspecific factors. Second, the sample size was relatively small (n=23). Third, the lack of 1-year follow-up data limits the generalizability of our conclusions. Larger- and longer-scale studies are necessary. Fourth, sleep estimates were based on subjective sleep diaries and PSQI scores, rather than on objective measures such as polysomnography. The use of both subjective and objective measures has been recommended [35,36]. Finally, Outcome assessors were not blinded; however, blinded outcome assessment is recommended in open label trials to reduce bias.

### Conclusions

In conclusion, our results suggested that a 6-week ICBT program is an effective treatment for patients with insomnia who remain symptomatic following pharmacologic treatment.

## Appendix

Multimedia Appendix 1 CONSORT-EHEALTH checklist (V 1.6.1).

## Abbreviations

ACP: American College of Physicians
ANCOVA: analysis of covariance
CBT: cognitive behavioral therapy
CES-D: Center for Epidemiologic Studies Depression Scale
CONSORT: Consolidated Standards of Reporting Trials
EQ-5D: EuroQol-5D
HADS: Hospital Anxiety and Depression Scale
ICBT: internet-delivered computerized cognitive behavioral therapy
NA: number of awakenings
PSQI: Pittsburgh Sleep Quality Index
QALY: quality-adjusted life year
QOL: quality of life
RCT: randomized controlled trial
SE: sleep efficiency
SOL: sleep onset latency
TST: total sleep time
UC: usual care
